# An online survey of chiropractors' opinions of continuing education

**DOI:** 10.1186/1746-1340-13-22

**Published:** 2005-10-21

**Authors:** Kent J Stuber, Jaroslaw P Grod, Dean L Smith, Paul Powers

**Affiliations:** 1 Private practice KS: Calgary, AB, Canada, DS: Eaton, OH, USA, PP: Rocky Hill, CT, USA; 2 Post-graduate faculty, University of Bridgeport Chiropractic College, Bridgeport, CT, USA; 3 Division of Continuing Education, Canadian Memorial Chiropractic College, Toronto, ON, Canada; 4 Department of Physical Education, Health and Sports Studies, Miami University, Oxford, OH, USA

## Abstract

**Background:**

Continuing Education (CE) for chiropractors is mandatory for licensure in most North American jurisdictions. Numerous chiropractic colleges have begun collaborating with universities to offer master's degree programs. Distance education master's degree programs may be desirable to allow full-time practicing doctors to further their post-graduate education. The present survey sought to answer three questions. First, what is the level of satisfaction of chiropractors with their continuing education? Second, what is the level of interest of chiropractors in online master's degree programs? Lastly, what is the response rate of chiropractors to an online survey?

**Methods:**

An online survey consisting of 22 multiple choice questions was e-mailed to 1000 chiropractors randomly selected from the mailing list of an online chiropractic newsletter. Upon completion of the questionnaire, participants' answers were saved on a secure site. Data analysis included evaluation of the demographic characteristics of the respondents, their opinions of and patterns of taking CE including online education, preferred learning formats, and their interest in proposed online master's degree programs. A survey response rate was determined.

**Results:**

Nearly 86% of respondents felt their previously completed CE courses were either somewhat or extremely satisfactory. Over ninety percent of respondents who had completed online or distance CE coursesfound them to be somewhat or extremelysatisfactory. Almost half the respondents indicated that they most preferred online distance learning, while 34.08% most preferred face-to-face interaction. Fifty-three percent of respondents indicated an interest in starting a master's degree program; however 70.46% of respondents were interested in an online master's degree program that would offer CE credit. A response rate of 35.8% was obtained.

**Conclusion:**

Satisfaction among chiropractors with CE programs is high. The notion of completing a part-time online master's degree (or online combined with face-to-face interaction) appears to be popular among respondents, with a M.Sc. in Chiropractic Sciences being the most popular of those mentioned. Online surveys are a viable method of obtaining opinion in a cost and time efficient manner; there are some sources of bias involved in this type of research, and numerous steps need to be taken to obtain a suitable response rate.

## Background

The objective of this study was to conduct an online survey of chiropractors to determine their satisfaction with current chiropractic continuing education programs and to ascertain their level of interest in a variety of online master's degree programs particularly online continuing education programs. This study also sought to assess the response rate of chiropractors to online surveys.

There is a paucity of information on opinions of chiropractors through survey studies regarding online education. The recent NBCE Job Analysis of Chiropractic [1] provides some minimal recent data. The input of practicing chiropractors as well as educators and regulators is important in developing and sculpting the future programs for chiropractors. Without appropriate surveys and environmental scans there is the danger of creating programs that are of no interest or practicality to field practitioners.

As with most health care professions, chiropractors in North America have mandatory continuing education requirements in most jurisdictions [2]. These continuing education requirements can be obtained through online continuing education courses, live seminars, workshops, and courses, and an assortment of other distance learning formats (telephone conferencing, text-based courses). In many provinces and states, continuing education courses are approved if offered or sponsored by a chiropractic college accredited by the Council on Chiropractic Education (CCE) or approved by the state/provincial regulatory board.

Many chiropractors choose to augment their professional education (Doctor of Chiropractic) with courses that can certify them as proficient in certain techniques or aspects of practice (such as the Certified Chiropractic Sports Physician certificate program). Others seek specialist level training, and this training can be obtained as either part of a 2–3 year residency program or in a part-time weekend course format over the course of three years (several of these programs now have distance learning components available as well). Organizations that offer these programs aim to provide more detailed or relevant information on topics such as: orthopedics, nutrition, neurology, pediatrics, etc. than an undergraduate chiropractic program. There is a problem with this state of affairs: most health care professionals (including chiropractors) do not know the value or significance of these "letters." It is common in the chiropractic literature to see numerous designations listed by an individual author – What does this mean to the reader? Is this person an expert? Is this person a specialist?

This trend has been called "credential inflation" [3] – many journals now only publish terminal degrees (MD, DC, PhD etc.), or none at all. Objectively assessing the expertise of doctors holding these certifications requires knowledge regarding the process of attaining the certification. The most common areas of certification include: orthopaedics, neurology, paediatrics, sports and rehabilitation. Some are related (e.g. CCSP leading to DABCSP) and some are proving difficult to track down.

It is fundamental to define what "chiropractic certification/specialty program" means. These are programs offered to chiropractors or chiropractic students who are enrolled in, or have graduated from, any accredited chiropractic college/university anywhere in the world. Upon completion of the program, the right to use the acronym of the particular specialty board, governing body, or program is granted to the doctor. There are numerous organizations within the chiropractic profession that offer these "specialty" certifications. Some are offered through chiropractic college or university settings, while others are offered through private organizations.

Chiropractic specialties are often compared to, and promoted as, the equivalent of medical specialties. This idea deserves further discussion, as certain standards should be met in order to legitimately achieve specialist standing. It is logical to assume that a specialist is someone who *practices a specialty after receiving advanced clinical training*. That is, someone who allots a significant portion of his or her practice to the specialty, and has significantly advanced knowledge and training in a particular area compared to an average chiropractor. This may only apply to a small number of chiropractic "specialties". These, along with other attributes of defining oneself as chiropractic "specialist", have been suggested by Nelson and Lawrence [3], and are listed in Table 1.

**Table 1:** Suggested Criteria to Define a Chiropractic Specialist

1) The training of a specialist should be substantially greater than that of the average chiropractor.
2) The training should consist primarily of actual patient contact, not simply repeated lectures (i.e. lectures they have already had in their undergraduate education).
3) A substantial part of the chiropractor's practice should be devoted to the specialty.
4) It should be possible to fail the certification process.
5) There should be some scholarly effort in the area of specialization: research, publication, or some other recognized contribution to the field.

*Adapted from: Nelson C & awrence DJ. Degree and certification proliferation and the JMPT. Journal of Manipulative and Physiological Therapeutics 1995; 18(2): 55–56*.

**Table 2:** Chiropractic Colleges, Partner Universities, Degrees and Study Format Offered.

**Chiropractic College**	**Partner University**	**Degree Offered**	**Study Format Offered**
New York Chiropractic College	None	Master of Science in Diagnostic Imaging	Full-time, on-site, residential program
	None	Master of Science in Acupuncture and Oriental Medicine	Full-time, on-site, residential program
	None	Master of Science in Acupuncture	Full-time, on-site, residential program
National University of Health Sciences	A.T. Still University	Master of Public Health (MPH)	On-line distance, part-time
Palmer College of Chiropractic	None	Master of Science in Anatomy	Full-time, on-site, residential program
	University of Iowa	Master of Science in Clinical Research	Full-time, on-site, residential program
Anglo-European Chiropractic College	University of Portsmouth	MSc in Advanced Professional Development in Chiropractic	Tutorials, workshops, seminars, practical skills classes, small group work, and lectures. Part-time.
		MSc in Advanced Professional Development in Chiropractic Pediatrics	Tutorials, workshops, seminars, practical skills classes, small group work, and lectures. Part-time.
Southern California University of Health Sciences	Royal Melbourne Institute of Technology University	Master of Applied Science in Musculoskeletal Management	Text-based distance, part-time. Distance education programs are designed as self-contained learning packages. Group sessions are scheduled periodically and communications are transmitted through e-mail and the internet.

Having said that, a master's degree offered by an accredited institution can potentially provide its graduates credentialed recognition (in areas such as health policy development, health administration, or research) and opportunities not available to those who complete a chiropractic specialty program. Chiropractors taking graduate-level courses from a non-chiropractic college or university are at times unable to obtain continuing education credit for their coursework. To counter this problem, numerous chiropractic colleges have begun either on their own or in collaboration with universities to offer master's degree programs. This collaboration allows chiropractors in these programs to obtain recognized graduate level education, training, and qualification while still obtaining continuing education credit (as the courses are offered by a chiropractic college). It is noteworthy to point out that master's degrees can be obtained in both chiropractic and non-chiropractic fields (see Table 2). Table 2 lists the chiropractic schools that are members of the Association of Chiropractic Colleges that currently offer a part-time master's degree program, their university partners, and the program and format offered at the time of publication.

Several of the programs listed in Table 2 require classroom interaction and instruction, and may be difficult for the practicing clinician to complete without taking extended time away from their practice. Predominantly distance learning master's degree programs may be desirable to allow full-time practicing doctors to obtain graduate level education. Numerous studies and reviews have found that online continuing education methods are at least as effective in increasing participant knowledge as traditional continuing education methods [4,5]. In fact, one recent randomized controlled trial found that evidence-based online continuing medical educationcan produce objectively measured changes in behavior as well as sustained gains in knowledge that are comparable or superior to those realized from effective live activities [6].

## Methods

An online survey consisting of 22 multiple choice questions was e-mailed to 1000 chiropractors (out of a pool of over 40,000) randomly selected by computer from the mailing list of ChiroWire. This is an online newsletter sent out by a continuing education provider for chiropractors in the United States and Canada. The survey was uniquely generated. Although the reliability of this instrument was not assessed, face and content validity was accepted by a panel of 3 chiropractors and a psychologist.

We surveyed 1000 chiropractors with the goal of achieving a sample size of 384. The target sample size was determined by using an alpha level of .05, with a conservative estimate of proportion (proportion of the population answering a particular way) equal to .50. We used the method proposed by Pittenger to calculate our survey sample size [7]. Hawk et al used a comparable method and determined that 380 chiropractors would be a desirable sample size for a similar survey [8]. Since we planned on using our results to describe chiropractors' opinions of CE as opposed to implementing a specific change in behavior with respect to CE, we were willing to tolerate a larger margin of sampling error [9].

At the time of conducting the study, it was not known what proportion of doctors on the mailing list had actually taken online CE programs in the past. This convenience sample of doctors was asked for their participation in the study by way of an e-mail message with the subject line of "Chiropractic continuing education study – please participate". The e-mail message consisted of an introduction, explaining the purpose of the survey, along with a link to an electronic informed consent form. After reading the introductory letter, the recipient could click on the link to the informed consent form if they wanted to participate in the study. The subject was then asked to read the informed consent form. If the subject consented to participation they clicked on an "I Consent" button at the end of the informed consent form that immediately linked them to a secure website consisting of the survey for completion (the secure page was hosted by ). If the subject did not consent to participation, they were asked to erase the message.

The email containing the link to the survey was sent to the database only once. No follow-up emails or other contact was attempted by the researchers. Doctors who responded within twelve days from the email were included in the survey results. Those who agreed to participate completed the 22 multiple choice questions (by simply 'pointing and clicking' on their answer) and then clicked on a "Submit" button upon completion of the survey. Results were stored by  until data analysis took place. Survey responses were anonymous and subjects were given the option of contacting the authors before or after completion of the study to have any questions answered. The online survey software has cookie technology and respondent session keys that prevented multiple responses from the same user. The questions in the survey consisted of demographic information, questions about preferred learning styles for continuing education, and which master's degree format and program content they would be most interested in. Ethics approval for this study was obtained from the University of Bridgeport Institutional Review Board.

Data analysis consisted of determining the demographic characteristics of the respondents, their opinions of and patterns of taking continuing education programs including online programs, and their interest in proposed master's degree programs and formats. A survey response rate was also determined.

## Results

### Demographics

Some respondents did not answer all of the questions; the number of respondents to several of the questions is indicated in the respective figures. Palmer College of Chiropractic alumni were the most common respondents (19.55%), followed by Life University (14.8%), National University of Health Sciences (10.34%), Logan College of Chiropractic (7.54%), Cleveland Chiropractic College (6.42%), Western States Chiropractic College (5.87%), and New York Chiropractic College (5.03%) (the preceding colleges had alumni who represented greater than five percent of respondents to this survey). The highest percentage of respondents had been in practice for 6–10 years (20.28%), followed closely by those in practice 16–20 years (19.44%), 21–25 years (18.03%), 11–15 years (17.46%), 0–5 years (16.34%), and finally by those in practice for 26 years or greater (8.45%).

Over two thirds (67.6%) of the respondents to this survey had a bachelor's degree, while 10.56% had master's degrees and 1.76% had a Ph.D. Nearly thirty percent (27.89%) of respondents have completed a diplomate program or equivalent indicating a practice specialty, and 10.42% were in the process of completing one.

### Continuing education opinions and practices

**Figure 1:**
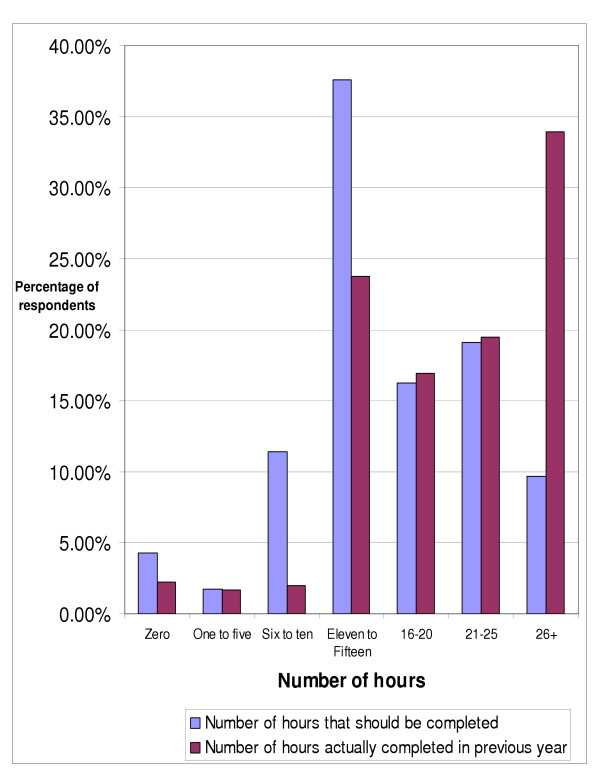
Opinion of number of hours of continuing education that chiropractors should be required to complete annually (n = 351) and number of hours actually completed by respondents (n = 354).

Nearly ninety-five percent (94.65%) of respondents indicated that they had mandatory continuing education requirements for re-licensure in their state or province, compared with 1.69% who indicated that continuing education was recommended, and 3.66% who indicated that their jurisdiction had no continuing education requirements. When asked how many continuing education hours they felt that chiropractors should have to complete annually, the most common response was eleven to fifteen hours by 37.61% of the respondents as seen in figure 1.

**Figure 2:**
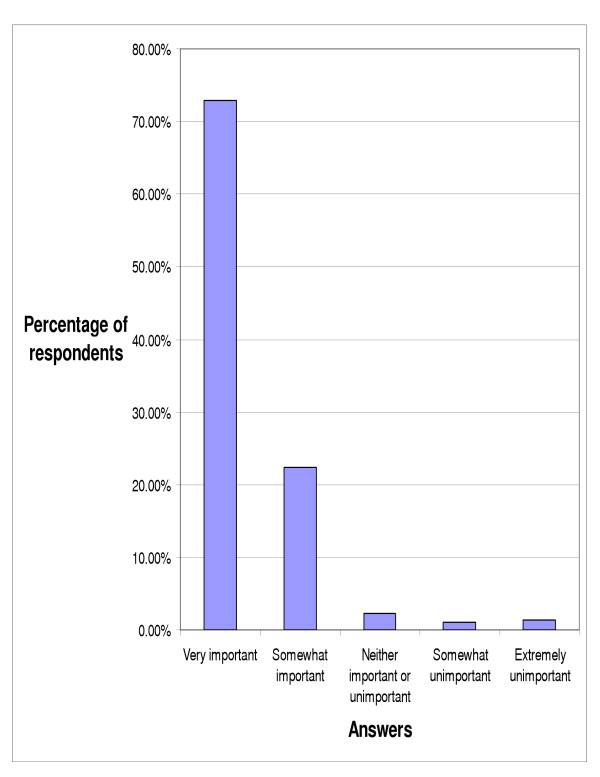
Opinion of importance of continuing education for chiropractors (n = 357).

**Figure 3:**
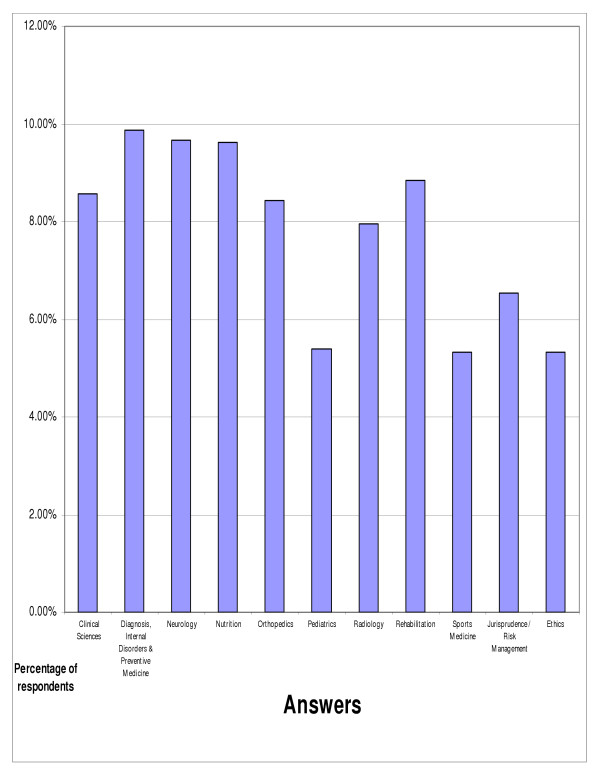
Areas of study that are most important for chiropractic continuing education (more than one answer could be selected) (only answers with greater than 5% response shown) (n = 2463).

With respect to the importance of chiropractic continuing education, greater than ninety five percent of responding chiropractors (95.24%) felt that CE was important, including 72.83% who felt that it was very important (figure 2). When asked which areas of study were most important in continuing education the doctors indicated that internal disorders & prevention (9.87%), neurology (9.66%), and nutrition (9.62%) were the three most important areas (figure 3). Additional topics of study were asked about, but we chose to report only those that were selected by greater than five percent of respondents (respondents could pick as many responses as they desired).

**Figure 4:**
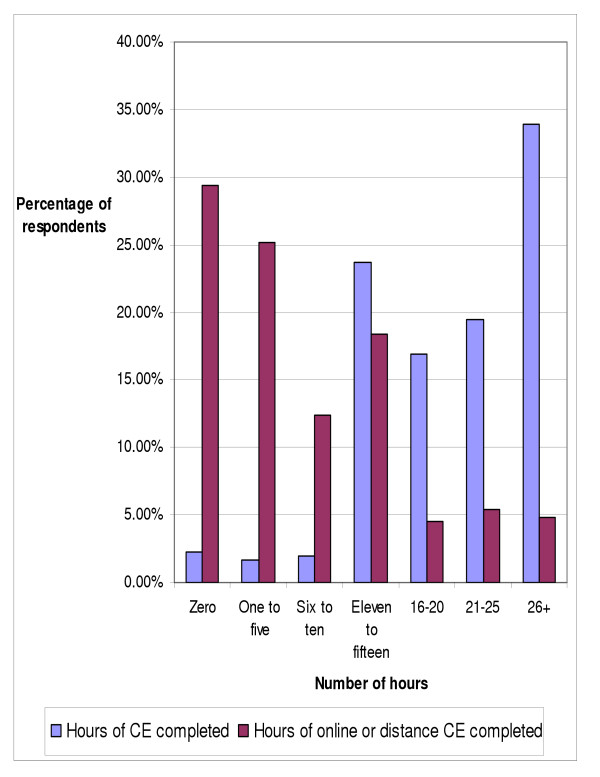
Number of hours of chiropractic continuing education and online or distance continuing education completed in the previous year (n = 354).

Figures one and four depict the number of continuing education hours completed in the previous year by the respondents, with greater than 26 hours being the most common response, by over one third of the doctors (33.9%). When asked about the number of online or distance continuing education hours completed in the previous year (figure 4), close to thirty percent (29.38%) of respondents did not do any online or distance continuing education, while approximately one in four (25.14%) completed one to five hours of online or distance CE.

**Figure 5:**
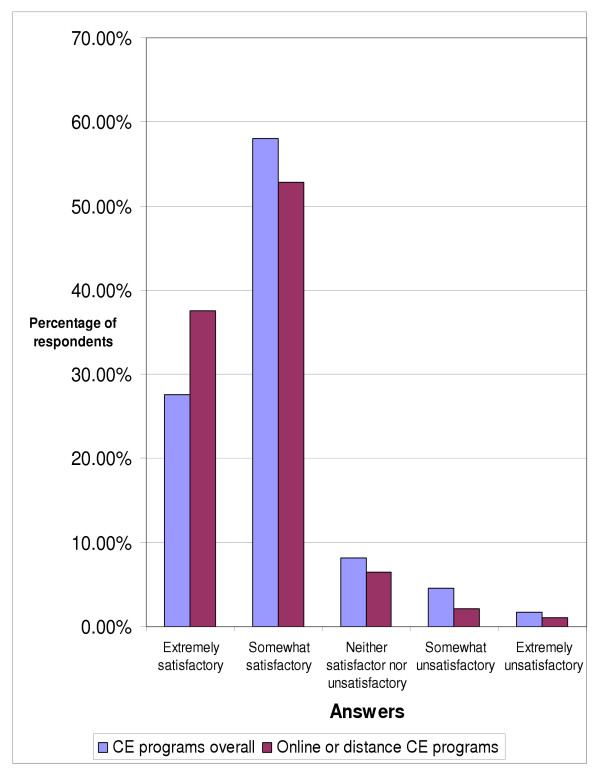
Opinion of completed continuing education programs, overall (n = 355) and online or distance (n = 280).

When asked for their opinion of continuing education courses that they had taken, over eighty percent (85.64%) of respondents replied that they were satisfactory (figure 5). Just over six percent (6.2%) of respondents felt that the courses they had completed were unsatisfactory. With respect to the doctors' opinions of online or distance continuing education courses that they had completed (figure 5) over ninety percent (90.36%) of those who had done some online or distance CE course responded that they were satisfactory. Slightly more than three percent (3.21%) felt that they were unsatisfactory. Over one in five respondents (20.45%) from the entire sample indicated that they hadn't taken any online or distance continuing education courses and could not comment.

When asked for their preference among different formats to fulfil their continuing education hours, close to fifty percent (46.48%) of the respondents indicated that they most preferred online distance learning, while over one third (34.08%) most preferred face-to-face interaction in the form of seminars, classes, and workshops. Nearly eighteen percent (17.75%) of the respondents indicated no preference among the different continuing education formats, while the remaining respondents preferred either telephone conferencing (0.28%) or text-based distance learning (1.41%).

**Figure 6:**
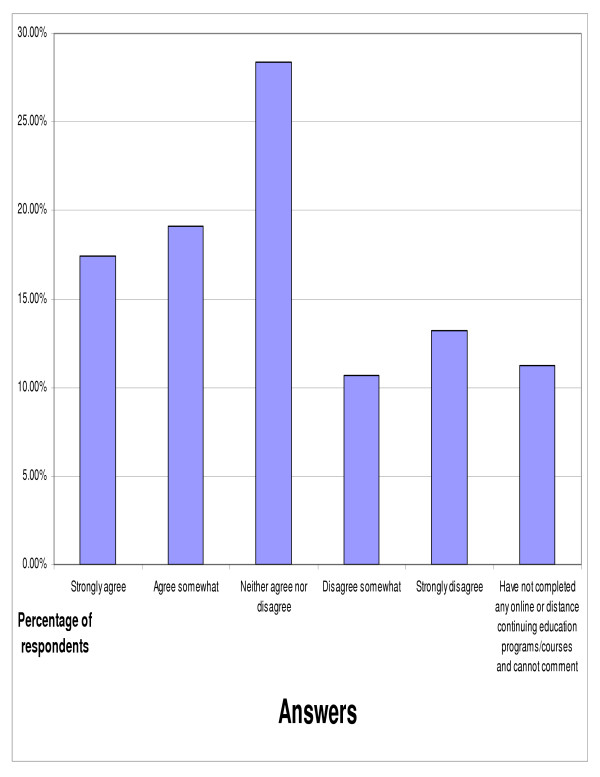
Response to the following statement, "One of the things that frustrates me with online or distance chiropractic continuing education programs is that I cannot get anything out of it to put on my resume like a diploma, etc." (n = 356).

The doctors were also asked for their opinions on the following statement, "One of the things that frustrates me with online or distance chiropractic continuing education programs is that I cannot get anything out of it to put on my resume, like a certificate, diplomate, or master's degree" (figure 6). Over one-third (36.52%) of respondents were in agreement with that statement. Nearly one quarter (23.87%) of respondents disagreed with the statement. Eleven percent of respondents indicated that they had not completed any online or distance continuing education programs and could not comment.

### Master's degree program interest

Over half (52.8%) of the respondents indicated an interest in starting a master's degree program; while over twenty percent (20.11%) of the respondents indicated that they were uninterested in a master's degree program. The remaining respondents either had no opinion one way or the other (21.51%), or were already in or had completed graduated studies (master's or PhD level) (5.59%). Nearly all of the respondents (96.91%) indicated being most interested in a part-time master's degree program in one form or another. This includes almost two thirds (63.16%) who indicated being most interested in a part-time online distance program, and nearly one third (31.27%) who indicated being most interest in a combination of online distance learning and face-to-face interaction in the form of seminars, classes, etc. Less than five percent (3.10%) of the respondents indicated that they were most interested in a full-time master's degree program.

Over forty percent (43.81%) of the respondents indicated being most interested in a Master of Science degree in Chiropractic Sciences, compared with 20.85% each for a Master of Science degree in Pain Management and a Master of Science in Rehabilitation. Close to fifteen percent (14.50%) of respondents indicated a preference in "Other" master's degree programs. We are unable to determine specifically what that may mean, other than those doctors were likely not interested in any of the three programs listed. Nearly eighty-five percent (84.59%) of the respondents indicated that if they were to take a master's degree program that they would rather take an elective course than complete a thesis or dissertation.

When asked if they would be interested in starting an online master's degree program with which they could obtain continuing education credit, over seventy percent (70.46%) of the respondents indicated an interest. One in eight (12.5%) respondents indicated that they were uninterested in such a program. The remaining 17.04% were neither interested nor disinterested (13.92%), or else they had already completed or were working on a master's degree or PhD (3.12%).

### Survey response rate

The response rate was calculated according to the following formula:

Calculated response rate = number of respondents/number of people who received the survey.

Calculated response rate = 358/1000 = 0.358 = 35.8%

Our final sample size of 358 was close to our target size of 384. Since we planned on using our results to describe chiropractors' opinions of CE as opposed to implementing a specific change in behavior with respect to CE, we were willing to tolerate a larger margin of sampling error [9]. Therefore, our final sample size was deemed satisfactory.

## Discussion

As stated earlier, the opinions of chiropractors surveyed assist in defining needs and developing the appropriate programs for improved clinical education. A patient-centred approach is both sought and preferred.

In the current NBCE survey (Job Analysis Survey NBCE 2005), 42.3% of chiropractors had practiced five to fifteen years [1]. The present study results show 37.74% of respondents who have been in practice from six to fifteen years, indicating that the results are similar. In 2003 the average chiropractor was in practice 15.6 years [1].

Nearly 60% (59.6%) of chiropractors have bachelor's degrees [1], the finding in the present study show a slightly higher number at 67.6%. The portion of chiropractors with bachelor's and master's degrees has increased [1]. Over six percent (6.3%) of chiropractors have master's degrees and 1.6% have doctorates in non-chiropractic fields [1]. Our results show that 10.56% of respondents have a master's degree and 1.76% have a Ph.D. The current results suggest that more chiropractors today have graduate level training than in the past. The two surveys (NBCE survey and our online survey) share similar results. The few minimal discrepancies between the two studies are most likely due to number of respondents and the resulting power of the studies. The NBCE Job Analysis surveyed 10,189 chiropractors out of a pool of 68,799 [1]. 3,370 (33.3%) responses were received [1]. In our study we received 358 (35.8%) responses from a pool of 1,000.

### Specialization

In the NBCE Job Analysis Survey, 64% of respondents had no diplomate status or equivalent specialty certification. The present survey obtained a considerably higher percentage of respondents who indicated diplomate status at 27.89%, compared with 14.4% reported previously [1]. Reasons for the difference between the present survey and the NBCE results are unknown.

### Continuing education

The 2005 NBCE Job Analysis Survey provided data regarding CE and other professional education activities. Most relevant to the present survey was that 97.1% of chiropractic practitioners continue their professional educational by attending conferences and seminars, 30% of chiropractic practitioners attend diplomate courses to continue their education, and 12.7% respondents indicated that they had participated on online credit courses [1]. The results from the present study show that 70.62% of respondents had completed some online or distance CE in the previous year, a much higher result than the NBCE survey.

The 2003 NBCE data indicates that chiropractors took more units of continuing education than the previous survey in 1998 [1]. One-fifth of respondents (19.4%) had completed 11–15 hours of CE, compared with 23.73% in the present study. In the NBCE survey, nearly one quarter (22.9%) of respondents had completed 16 to 20 hours, slightly higher than the 16.95% in our survey. One-fifth (21.6%) of NBCE respondents had completed 21 to 25 hours, comparable with our result of 19.49%. Almost thirty percent (29.2%) of NBCE respondents had completed 26 or more hours, a result similar to our 33.9%.

The results of this survey indicate that chiropractors overwhelmingly feel that continuing education is important. This agrees with Bolton's findings that chiropractors have a positive attitude towards continuing professional education along with continuing professional development and are aware of the need to keep current on new knowledge and technology [10]

The most popular choice among the respondents in this survey for the number of annual continuing education hours that chiropractors *should *complete was eleven to fifteen hours. Interestingly, the most frequently indicated choice for the number of continuing education hours *actually *completed in the previous year was twenty-six hours or greater. These results are difficult to compare with Bolton's findings that 40.1% of surveyed chiropractors regularly attended continuing professional education/development (CPE/D) courses and 52.6% occasionally attended CPE/D courses, while 7.3% never attended CPE/D [10]. Certain areas of continuing education study appear to be considered more important by the respondents, particularly internal disorders and prevention, neurology, nutrition, rehabilitation, clinical sciences, and orthopedics.

Among those indicating a preference, online distance was favored most by those surveyed, followed by face-to-face seminars and classes. These results differ from Cobb's review of online continuing health professional education and Stancic et al's study where both studies found that in-person continuing education was the most popular format of continuing education learning over online learning, although it was noted that the popularity of online formats was increasing [4,11]. In contrast, our sample would prefer to take online CE compared to other formats such as live, face-to-face interaction. These results indicate that chiropractic colleges and continuing education providers certainly should consider offering online distance education programs, but traditional face-to-face seminars, conventions, and classes should still be offered as well.

Nearly 30% of those surveyed indicated that they had not completed any online continuing education in the previous year, while approximately one in four indicated that they completed one to five hours of online continuing education in the past year (meaning that close to 55% of respondents completed five hours or less of online continuing education in the previous year). This result is lower than the results from Casebeer et al's survey of physicians that found that 70% of those surveyed rarely or never accessed CME online, perhaps indicating that chiropractors access online continuing education courses more than physicians [12].

While online continuing education appears to be popular among chiropractors, most of the doctors are not completing tremendous numbers of online continuing education hours. This is demonstrated by the fewer than 15% of the doctors who indicated that they completed sixteen or more hours of online continuing education in the previous year. Stancic et al found that there were four factors that helped a physician to determine whether they take a continuing education program: cost, personal control over content, personal development, and convenience of access [11]. Casebeer et al found that physicians desire online professional development courses that are easy-to-use, relevant, valid in content, credible, and available for immediate access [12]. Online continuing education providers need to design courses with these factors and wishes in mind, as these qualities are likely important to all groups of health care professionals.

A very strong majority (>85%) of the chiropractors surveyed appear to be satisfied with the continuing education courses that they have completed thus far. Another strong majority (>90%) of the chiropractors indicated satisfaction with online or distance continuing education courses they had completed. This is in agreement with what Cobb found in her literature review of online continuing health education, in that health care professionals were satisfied with computer-based continuing education programs that they had participated in [4].

Of those who had completed online continuing education nearly 37% agreed with a statement indicating frustration with not obtaining certificates, diplomate status, or a master's degree from online or distance continuing education programs. This would seem to indicate that those offering online CE programs should consider the possibility of offering diplomate, master's degree, or certifications.

The results of this survey indicate that the chiropractors who responded generally appear to have an interest in completing a master's degree program (greater than 50%). This number increases to over 70% if the proposed program would be online and count towards continuing education credit, including greater than one in three respondents who were very interested such a program. There is also much greater interest from respondents in part-time master's degree programs than full-time programs, particularly if those part-time programs are either entirely or partially online. This is not an unexpected result as it is likely that most of the chiropractors completing our survey are in full-time practice (although we did not ask this question of respondents) and leaving practice to pursue a master's degree full-time may not be feasible. A part-time completely online master's degree program was of greatest interest for respondents, being preferred at a rate of over 2 to 1 to a program that would combine online learning with face-to-face interaction in the form of seminars or classes.

An M.Sc in Chiropractic Sciences was preferred at a rate of greater than 2 to 1 when compared to either an M.Sc in Pain Management or an M.Sc in Rehabilitation, for whom doctors had equal preference. The respondents in this survey showed a greater preference towards completing elective courses in a master's degree program when compared to completing a thesis or dissertation. This is not surprising as it is most likely that field practitioners may not have the interest or time necessary for conducting the research needed to be done to complete a thesis or dissertation. A question that a follow-up study could consider is which areas would be of most interest for an elective course.

### Online survey response

The calculated response rate for this study was 35.8%. This is within the range reported by Russell et al (7.0% to 91.4%) for surveys of chiropractors [13]. However, this is not an extremely high response rate and thus a potential source of bias due to non-response. There are numerous ways that the response rate in this study could have been improved. First, the survey was only sent to the sample population once. Sending the survey numerous times or sending out reminders out to those sampled may have improved the response rate, as mentioned by Russell *et al *[13]. Use of advance notice is also known to lead to higher response rates. We did not employ this technique.

An additional issue in this study is that the initial e-mail inviting each doctor to participate was sent to 1000 random chiropractors out of a pool of 40,000. A blinded randomizing program was used to select those who received the e-mail containing the survey and thus it could not actually be ascertained as to who received e-mails and who did not. This would make it extremely difficult to re-send the survey or send a reminder. It is possible to create a blinded program that would randomly select those to be surveyed and send them an initial e-mail as advanced notice of the survey, followed by an e-mail containing the survey; followed by periodic e-mails containing reminders and/or additional copies of the survey. However, this was not done in this study. Finally, by only allowing 12 days for subjects to respond to the survey before tallying the data, this study may not have yielded the optimal number of responses. A longer period for responses combined with advanced notice, reminder e-mails and/or additional copies of the survey may allow for higher response rates.

Online surveys are a potentially attractive option for opinion based survey research in the future. They have the potential to be cost-effective, as the costs of postage (and return postage) and copying is not a factor. However, in the case of this survey, a third-party had to be contracted to construct the survey (after they were provided with the questions) and to receive and tally the results from the subjects. It is possible that this could be done from a single e-mail address or web page set up by the authors, but having a service perform this function worked in this case and the cost involved was reasonable, certainly less than the above-mentioned costs for postage and copying. Online surveys can also be sent out and responses received quickly. As mentioned previously, this study obtained 358 responses in 12 days; this would take far longer using conventional postal surveys.

One potential drawback or source of bias in online surveys is that they can only be taken by internet capable individuals. It is possible that older doctors or other doctors who do not frequently use the internet or do not have internet access would not be able to be surveyed in this manner. Another potential source of bias in this study is that the sample came from a group of chiropractors who signed themselves up with an online continuing education company. It is reasonable to assume that these individuals may be more interested in online continuing education than other doctors who have not signed up with such a service.

A final source of bias is that the online continuing education company whose subscribers were surveyed is an American company, and this could explain the small numbers of Canadian and international doctors responding (4.23% and 3.10%, respectively). As mentioned previously, some respondents did not answer all of the questions, and so the complete results from those surveyed were not obtained. It is unknown as to why some doctors chose not to answer some questions.

Future studies could delve into whether there is a difference in continuing education patterns and opinions between Canadian and American doctors, and graduates of different schools.

## Conclusion

The satisfaction rate among chiropractors taking CE programs (including online programs) is high. The notion of completing a part-time online master's degree (or online combined with in class sessions) appears to be popular among respondents, with a M.Sc. in Chiropractic Sciences being most popular among respondents. Finally, online surveys are a viable method of obtaining opinion in a cost and time efficient manner. There are some sources of bias (e.g. sampling bias) involved in this type of research, and numerous steps need to be taken to obtain a suitable response rate.

Chiropractors feel that continuing education is important. Among chiropractors surveyed, online CE is the most preferred method of learning over live lectures or workshops, and other methods of distance learning. Certain areas of chiropractic CE appear to be more important to those surveyed, particularly internal disorders and prevention, neurology, nutrition, rehabilitation, clinical sciences, and orthopedics.

## Competing interests

KJS: post-graduate online instructor for chirocredit.com, an online continuing education provider sponsored by the University of Bridgeport Chiropractic College, author of an online continuing education course for Canadian Memorial Chiropractic College's Division of Continuing Education.

JPG: Director of Canadian Memorial Chiropractic College's Division of Continuing Education

DS: post-graduate online instructor for chirocredit.com, an online continuing education provider sponsored by the University of Bridgeport Chiropractic College

PP: post-graduate online instructor for chirocredit.com, an online continuing education providersponsored bythe University of Bridgeport Chiropractic College; CEO of OnlineContinuingEd, LLC, anonline continuing education provider.

## Authors' contributions

KJS was involved in study conception, study design, data analysis and interpretation, and manuscript preparation and revision. JPG carried out data analysis and interpretation, and was involved with manuscript preparation and revision. DLS took part in study conception, study design, and manuscript preparation and revision. PP was involved with study conception, study design, data acquisition, and manuscript revision. All authors read and approved the final manuscript.

## References

[B1] Christensen M, Kollasch M (2005). Job Analysis of Chiropractic.

[B2] Pallister S (1989). Continuing education for chiropractors in Canada. J Can Chiro Assoc.

[B3] Nelson C, Lawrence DJ (1995). Degree and certification proliferation and the JMPT. Journal of Manipulative and Physiological Therapeutics.

[B4] Cobb SC (2004). Internet continuing education for health care professionals: an integrative review. J Contin Educ Health Prof.

[B5] Wutoh R, Boren SA, Balas EA (2004). E-learning: a review of internet based continuing medical education. J Contin Educ Health Prof.

[B6] Fordis M, King JE, Ballantyne CM, Jones PH, Schneider KH, Spann SJ, Greenberg SB, Greisinger AJ (2005). Comparison of the instructional efficacy of internet-based CME with live interactive CME workshops: a randomized controlled trial. J Am Med Assoc.

[B7] Pittinger DJ (2003). Behavioral Research Design and Analysis.

[B8] Hawk C, Dusio ME (1995). A survey of 492 US chiropractors on primary care and prevention-related issues. Journal of Manipulative and Physiological Therapeutics.

[B9] Bordens KS, Abbott BB (2002). Research Design and Methods: A Process Approach.

[B10] Bolton JE (2002). Chiropractors' attitudes to, and perceptions of, the impact of continuing professional education on clinical practice. Med Educ.

[B11] Stancic N, Mullen PD, Prokhorov AV, Frankowski RF, McAlister AL (2003). Continuing medical education: what delivery format do physicians prefer?. J Contin Educ Health Prof.

[B12] Casebeer L, Bennett N, Kristofco R, Carillo A, Centor R (2002). Physician internet medical information seeking and on-line continuing education use patterns. J Contin Educ Health Prof.

[B13] Russell ML, Vehoef MJ, Injeyan HA, McMorland DG (2004). Response rates for surveys of chiropractors. J Manipulative Physiol Ther.

